# A graphical method for practical and informative identifiability analyses of physiological models: A case study of insulin kinetics and sensitivity

**DOI:** 10.1186/1475-925X-10-39

**Published:** 2011-05-26

**Authors:** Paul D Docherty, J Geoffrey Chase, Thomas F Lotz, Thomas Desaive

**Affiliations:** 1 Centre for Bioengineering, Department of Mechanical Engineering, University of Canterbury, New Zealand, Private Bag 4800, Christchurch, New Zealand; 2 Cardiovascular Research Centre, institute of Physics, University of Liege, Belgium, Allée du 6 Août, 17 (Bât B5), B4000 Liege, Belgium

## Abstract

**Background:**

Derivative based a-priori structural identifiability analyses of mathematical models can offer valuable insight into the identifiability of model parameters. However, these analyses are only capable of a binary confirmation of the mathematical distinction of parameters and a positive outcome can begin to lose relevance when measurement error is introduced. This article presents an integral based method that allows the observation of the identifiability of models with two-parameters in the presence of assay error.

**Methods:**

The method measures the distinction of the integral formulations of the parameter coefficients at the proposed sampling times. It can thus predict the susceptibility of the parameters to the effects of measurement error. The method is tested *in-silico *with Monte Carlo analyses of a number of insulin sensitivity test applications.

**Results:**

The method successfully captured the analogous nature of identifiability observed in Monte Carlo analyses of a number of cases including protocol alterations, parameter changes and differences in participant behaviour. However, due to the numerical nature of the analyses, prediction was not perfect in all cases.

**Conclusions:**

Thus although the current method has valuable and significant capabilities in terms of study or test protocol design, additional developments would further strengthen the predictive capability of the method. Finally, the method captures the experimental reality that sampling error and timing can negate assumed parameter identifiability and that identifiability is a continuous rather than discrete phenomenon.

## 1. Background

A number of physiological phenomenon have been modelled by formulating mathematical representations of the relevant interactions. These models frequently incorporate variable parameters that can be identified to match the model representation to the observed behaviour. The value of these parameters is then used to characterise or quantify the response. However, with complex or large models, variable parameters can be selected that seem mathematically distinct, but in reality define the same observable effect and identification failure is certain. Thus, model identifiability analyses are used to test the selection of model parameters and ensure that they are mathematically distinct.

Approaches for the analysis of model identifiability typically assume continuous perfect input data [1-3]. However, these derivative-based identifiability methods can produce false assurances of identifiability. Recent dissatisfaction with the classical algorithms has resulted in the development of new methods that recognise assay error and discrete measurements as critical to identifiability [4,5]. The limitation of discrete data that is often subject of assay error often causes parameter trade-off [6-9] and thus limitation of the identified metrics clinical value. Thus, not only should a model be checked for identifiability in the classical a-priori sense, but the susceptibility of parameters to mutual interference should also be tested.

For example, the Minimal Model of insulin sensitivity [10] has been shown to be identifiable using such methods [11-13]. However, with discrete data that is subject to assay error, parameter identification has sometimes failed [6,7,14]. Numerous Bayesian techniques have had success in limiting this failure [7,15-17], but they tend to force the parameters to diverge away from their true least square values, limiting the relevance of the model and exaggerating the influence of population trends on an individual test's identified parameter values. Thus, widespread clinical application of these models has been limited by the ambiguity of results.

This article presents a novel graphical method for identifiability analysis that allows an identifiability analysis with consideration of noise and assay error. Furthermore, the method highlights areas for potential improvements to protocols and sampling times that would improve practical identifiability. At this stage of development, the method is limited to first-order, two-parameter models that allow a separation of parameters, but are typical of those found in pharmacokinetic (PK) and pharmacodynamic (PD) modelling.

## 2. Method and Study Design

The proposed method will be evaluated *in-silico *using clinically validated models of insulin kinetics and the dynamic between insulin concentration and glucose decay. The method's ability to predict the variation behaviour of identified parameters in a Monte Carlo analysis will be tested.

### 2.1 Proposed method process

To evaluate identifiability of a model, the integral formulations of the parameters are evaluated using an estimated test stimulus response. Thus, the method cannot be used in complete ignorance of the expected behaviour of the test participant. In particular, the approximate shape of the species concentrations as a result of the test protocol must be estimated, (this is a reasonable assumption in most PK/PD studies). The specific steps are illustrated using a generalised function:(i)

where: *X *is a measured species with a discrete resolution, *Y *is a species that is co-dependent with *X *and is not measureable, *C *and *D *are independent known input profiles, and *a *and *b *are unknown model parameters

1. Rearrange governing equation to create a first order differential equation with separated parameters in terms of a-priori, constant and measurable concentration terms(ii)

2. Derive the integral formulation of this governing equation(iii)

where: *i *is each measured sample time after the first.

3. Evaluate the integral of the coefficients of each parameter between 0 and each proposed sample time using an assumed participant response to the test stimulus.(iv)

4. Divide the resulting values by their respective means to normalise the coefficients.(v)

5. Subtract one set of coefficients from the other and define the 2-norm of the result (||Δ||_2_).(vi)

6. Any distinction at all between the coefficients would imply identifiability (i.e. if ||Δ||_2 _≠ 0). In reality, the effect of assay error on parameter identification is inversely proportional to the magnitude of this distinction (and proportional to the magnitude of any assay error (*ε*)):(1)

where: *μ *is a proportionality factor that incorporates factors such as the relative contribution of the parameter to the derivative of the relevant species concentration in the governing differential equation, and the absolute magnitude of the noise at the sampled times in relation to the relative magnitude of the parameter coefficient.

Thus, the method cannot accurately predict the coefficient of variation that a Monte Carlo analysis may find. However, it can predict the change of variation that might be observed when changes are made to the test sampling or stimulus protocols.

### 2.2 The dynamic insulin sensitivity and secretion test (DISST) model

#### 2.2.1 Insulin pharmacokinetics

Initially, a validated model of insulin PKs [18,19] is used to evaluate the method described, and is defined:(2)(3)

where: equation nomenclature is defined in Table 1

**Table 1:** Nomenclature from Equations 2 and 3

Symbol	definition	units
*I*	Plasma insulin concentration	mU/L
*Q*	Interstitial insulin concentration	mU/L
*U_N_*	Endogenous insulin production rate profile	mU/L/min
*U_X_*	Exogenous insulin bolus	mmol
*n_K_*	Renal clearance of plasma insulin	1/min
*n_L_*	Hepatic clearance of plasma insulin	1/min
*n_C_*	Insulin clearance to cells	1/min
*n_I_*	Transition of insulin between plasma and interstitium	L/min
*α_I_*	Saturation of hepatic insulin clearance	L/mU
*x_L_*	First pass clearance of insulin	1
*V_P_*	Distribution volume of plasma insulin	L
*V_Q_*	Distribution volume of interstitial insulin	L

The model is used in the dynamic insulin sensitivity and secretion test (DISST) to define the PKs of insulin due to test stimulus [18,20]. The model assumes that plasma insulin (*I*) is sampled. A traditional derivative based identifiability analysis of the model presented in Equations 2 and 3 using Ritt's pseudo-division algorithm [1,21] is presented in Appendix 1.

#### 2.2.2 Pharmaco-dynamics of glucose and insulin

The parameters of the glucose-insulin PDs can also offer insight into the identifiability of parameters. The DISST model of glucose-insulin PDs is defined [18,19]:(4)

where: equation nomenclature is defined in Table 2.

**Table 2:** Nomenclature from Equation 4

Symbol	definition	units
*G*	Glucose concentration	mmol/L
*Q*	Interstitial insulin concentration	mU/L
*G_b_*	Basal glucose concentration	mmol/L
*Q_b_*	Basal interstitial insulin concentration	mU/L
*p_G_*	Glucose dependant glucose clearance	1/min
*S_I_*	Insulin sensitivity	L/mU/min
*P_X_*	Exogenous glucose bolus	mmol
*V_G_*	Glucose distribution volume	L

Although the structural identifiability of Equation 4 is trivial it is also presented in Appendix 1.

### 2.3 Participants

Parameter values from two participants of the pilot investigation of the DISST [18] are used to generate *in-silico *simulated data to construct and demonstrate the method proposed here. *In-silico *data is used in this analysis because it allows protocols to be changed to illustrate the impact on identifiability. The participant characteristics that are summarised in Table 3 represent the extremities of the range of cases encountered in typical research studies of insulin sensitivity.

**Table 3:** Anatomical and identified parameter values

Glucose tolerance	Sex	Age	BMI	*U_N_*^†^			I clearance		*V_G_*	*SI*
						
				*U_b_*	*U_1_*	*U_2_*	*n_L_*	*x_L_*		
**NGT**	M	22	21.5	26.6	487.7	9.7	0.218	0.797	9.75	20.95
IGT*	F	57	33.9	115.5	233.6	150.7	0.064	0.822	13.35	2.236

Anatomical and identified parameter values (using the iterative integral method) of normal-glucose tolerant (NGT) and impaired glucose tolerant (IGT) participants of the DISST pilot investigation. (* The IGT participant had suspected, yet un-diagnosed, type 2 diabetes at the time of testing, ^† ^*U_b_, U_1 _*and *U_2 _*represent the basal, first and second phases of insulin production, respectively [mU/min])

### 2.4 Simulated test protocol

The simulated protocol is similar to the DISST test; a 10 g glucose bolus is administered at *t *= 7.5 and a 1 U insulin bolus at *t *= 17.5. The test duration is 60 minutes with a 5 minute sampling frequency. The *U_N _*profile is defined as a step function with three stages including basal, first and second phase production rates. The first phase of insulin production has a five-minute duration and begins with the glucose bolus. Simulations of plasma and interstitial insulin are completed using Equations 2 and 3, the parameter estimation equations from [22], *n_L _*and *x_L _*values from Table 3 and an *a_I _value of 0.001 L/mU*. Glucose is simulated using Equation 4 and the interstitial insulin profile obtained in the evaluation of Equations 2 and 3.

### 2.5 Iterative integral method

The analyses of this study will use the iterative integral method to identify parameters [20,23]. Although the method has been presented [20], it is repeated in brief in Steps 1-5 below:

1. The method converts the model governing equations to their integral formulation.

2. The parameter coefficients and the remainder terms are evaluated between *t *= 0 and the sample times.

3. The coefficients form the LHS of a matrix equation in terms of the parameters, while the remainder terms form the RHS. This matrix is evaluated to get parameter values.

4. These parameter values are used to update the coefficient evaluations in step 2, which enables a more accurate matrix equation in step 3.

5. Step 4 is iterated until convergence is achieved.

### 2.6 Analysis

A series of parameter and sampling scenarios were analysed using the models presented in a Monte Carlo analysis. Clinically measured physiological parameters of the NGT participant presented in Table 3 were used to define simulated responses to the test protocol described in Section 2.4. Samples were obtained from the simulated profiles at the defined times. Each iteration of the Monte Carlo analysis adds normally distributed assay error (the error magnitude is defined within each section). 100 iterations are used for each analysis with parameter identification by the iterative integral method. Only simulated profiles from the NGT participant are used until Section 3.2.2. Each scenario will present the mean value of those identified in the Monte Carlo simulation normalised by the simulation value from Table 3, and the coefficient of variation (CV) of each identified parameter (i.e. A mean of 1 and standard deviation of 0 implies perfect identification). The CV values are the paramount indicator of parameter trade-off during identification. The CV values are thus was compared to the *ε*/||Δ||_2 _value defined using the methods of Section 2.1 to obtained values for *μ *that linearise Equation 1.

### 2.7 Cases tested

A series of indicative cases will be investigated to show how the selection of model parameters, sample placement, participant behaviour and protocol dosing can have an effect on model identifiability.

• The insulin pharmacodynamic model will include five variable parameters to be identified to confirm the robustness and convexity of the iterative integral method, and to confirm the findings of the traditional derivative-based identifiability analysis.

• Parameter interference of *n_L _*and *n_K _*will be analysed. The nature of identifiability will be explored by alteration of the *α_I _*term that moderates the denominator of *n_L _*in Equation 2.

• The effect of sample selection on parameter identification of *n_T _*and *V_P _*will be measured. (*n_T _*is the addition of *n_L _*and *n_K_*).

• The effects of sample omissions on *SI *and *V_G _*identification are measured.

• The disparity of parameter identifiability in insulin resistant and sensitive individuals will be assessed using *p_G _*and *SI *as variable model parameters.

• The protocol proposed in Section 2.4 will be altered to see if the identifiability of insulin resistant participants can be improved.

• Measured samples that the proposed method claims are not valuable to stable identification are ignored and the identification process is repeated to confirm the prediction.

All cases will be tested with 1% and 3.5% normally distributed noise added to the virtually obtained data (to a maximum of 3 standard deviations).

## 3. Results

### 3.1 Analysis of the insulin pharmacokinetic model

#### 3.1.1 Confirmation of global model identifiability

**Figure 1:**
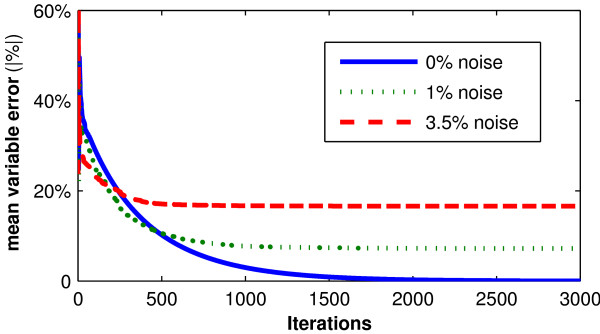
**Convergence of 5 parameter case**. Mean absolute percentage error between the simulation and identified parameters in the presence of 0%, 1% and 3.5% random assay error, with respect to iterations of the iterative integral identification approach.

When the sampled data is used in the iterative integral method to define *n_L_*, *n_K_*, *n_I_*/*V_P_*, *x_L _*and *V_P _*as parameters, convergence to the simulation values occurs (Figure 1). This confirms the traditional identifiability analysis of Equations 2 and 3 in Appendix 1. However, when 1% normally distributed noise is added to the simulated data, parameter values do not converge to simulation values (Figure 1). When the sample noise is increased to 3.5%, which is more indicative of actual measurement noise encountered clinically, parameter convergence is significantly biased. Hence despite proven (no noise) structural identifiability, the addition of assay error or noise yields corrupted or potentially unidentifiable results.

#### 3.1.2 Hepatic and renal clearance rate identification

To understand why the addition of noise disables the identification, the case of interference between *n_K _*and *n_L _*is tested. In this analysis, all parameters of Equations 2 and 3 are set as constants and only *n_L _*and *n_K _*are identified as parameters. From the analyses in Appendix 1 and Section 3.1.1, parameter convergence is assured for the noiseless case. However, for the 1% and 3.5% noise cases parameter interference causes considerable parameter divergence in the value of the identified parameters compared to the actual values used *in-silico*. The Monte Carlo analysis described in Section 2.6 is used to evaluate these parameters in the presence of noise and the results are shown in Table 4.

**Table 4:** Variation in identified *n_K _*and *n_L _*values

Noise	0%		1%		3.5%	
Saturation	*α_I _*= 0.001	*α_I _*= 0.05	*α_I _*= 0.001	*α_I _*= 0.05	*α_I _*= 0.001	*α_I _*= 0.05
***n_K_***	1(0)	1(0)	0.952(0.297)	0.999(0.017)	0.946(0.836)	1.004(0.059)
***n_L_***	1(0)	1(0)	1.035(0.214)	1.001(0.008)	1.041(0.595)	1.000(0.028)

The normalised parameter variation (mean(CV)) when *n_K _*and *n_L _*are identified model parameters with distinct simulated assay error and values for *α_I_*. (1(0) indicates perfect convergence to the simulation value)

The matrix equation used by the iterative integral method is a re-arrangement of Equation 2 and is in the form:(2a)

where: *i*= 5, 10, 15, ..., 60 minutes, matching sampling times.

Thus, the value of the ||Δ||_2 _term can be obtained for this model and sampling protocol as:

However, Table 4 shows that if *a_I _*is increased significantly to 0.05 L/mU, an arbitrarily chosen value that is not necessarily representative of physiology [24], parameter convergence is more stable. The ||Δ||_2 _term can be re-identified with the exaggerated *α_I _*value.

Thus, the parameters identified with the exaggerated *α_I _*term should have approximately 15 times smaller variability than those identified with the accepted *α_I _*value. Table 4 shows the effect of the *α_I _*distinction on the identified parameter values.

Table 4 shows the distinction between the effects of noise on identified parameters when the *α_I _*term is changed. Although the 0% noise case indicates that the parameters are uniquely identifiable, at 1% noise the variation in the identified values limits their clinical viability. At 3.5% noise, which may be expected in a real clinical setting, the parameters are no longer uniquely identifiable. This is illustrated by the very large CVs of the parameters. However, when the *α_I _*term is significantly increased, unique identifiability is once again possible, even with 3.5% noise. The mean ratio of variation caused by the disparate *a_I _*values was approximately 1:20. This is larger than the ratio predicted by the method (1:15), but still represents a positive outcome in terms of predicting the relative magnitude of the change.

**Figure 2:**
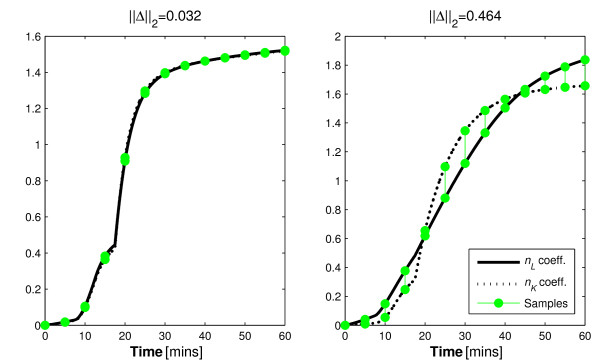
**Renal and hepatic clearance coefficient integrals**. The distinctions between the integral form of the hepatic and renal clearance coefficients when the standard (left) and exaggerated (right) *α_I _*values are used.

The reason for this outcome can be observed in the increased contrast between integral formulations of the parameter coefficients. The contrast is shown graphically in Figure 2.

The difference between the curves at the sample times indicates the identifiability of the model parameters in this two-parameter case. Thus, when the saturation term is increased, the coefficients of the parameters are more distinct and identifiability is increased. Despite the positive findings of the typical identifiability analysis, a saturation value of 0.001 L/mU causes *n_K _*and *n_L _*to become uniquely un-identifiable in a real clinical setting. This outcome may be considered as an elementary finding that should be inferred with a quick observation of Equation 2. However, it points to a failing of typical a-priori identifiability tests that this approach can negate with a quick graphical analysis.

The findings of this analysis also show that the functional effects of *n_L _*and *n_K _*on insulin concentration in Equation 2 are so similar that there would be a negligible effect if the terms are combined. As such, further analysis of this model will use a combined *n_L _*and *n_K _*term (*n_T_*) without the saturation term, which is negligible except at extremely high insulin concentrations. Equation 2 is thus redefined in Equation 5:(5)

#### 3.1.3 Plasma insulin distribution volume and insulin clearance identifiability

**Figure 3:**
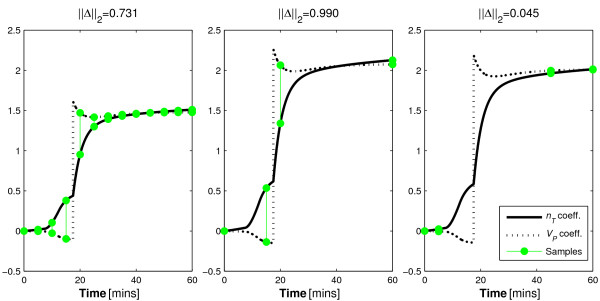
**Plasma distribution volume and inuslin clearance coefficient integrals**. Parameter coefficient distinctions using the three similar clinical protocols with distinct sampling regimens.

To identify *n_T _*and *Vp *the form of the governing matrix equation is:(5a)

where: 

As with the *n_K _*and *n_L _*analysis, the 0% noise case exactly reproduced the simulation values. Figure 3 shows the coefficients of the two parameters and three sampling protocols. In this case, Protocol 1 uses the 5 minutely sampling defined in Section 2.4. However, Protocol 2 uses samples at *t *= 0, 15, 20, and 60, and Protocol 3 uses samples at *t *= 0, 5, 45, and 60 minutes. Thus, Protocol 1 requires 13 samples while both protocol 2 and 3 only require four samples.

The ||Δ||_2 _terms can be defined for each of these protocols (Table 5) and the coefficients are displayed graphically in Figure 3.

**Table 5:** ||Δ||_2 _values from three sampling protocols defined in Section 3.1.3

	**||Δ||**_**2**_
**Protocol 1**	0.731
**Protocol 2**	0.990
**Protocol 3**	0.045

The ||Δ||_2 _values indicate that Protocol 3 will be comparatively unable to reproduce the simulation values. Contrary to the expected result that parameter identification is best with frequently sampled Protocol 1, the method predicts that the sparsely sampled Protocol 2 will be slightly more accurate.

Table 6 shows the parameter convergence and variability for the exact same model with the three different sampling protocols.

**Table 6:** Protocol dependence of identified *n_T _*and *V_P _*values

Noise	1%			3.5%		
Protocol	1	2	3	1	2	3
***n_T_***	1(0.004)	1(0.003)	0.973(0.012)	1.003(0.012)	1.001(0.014)	0.958(0.045)
***V_P_***	1.001(0.014)	0.995(0.012)	1.231(0.175)	0.999(0.047)	0.995(0.051)	1.190(0.463)

Normalised mean and (CV) of the identified values.

It is evident that although Protocols 2 and 3 contain the same number of samples, the resolution of the identified parameters is considerably reduced in Protocol 3. In effect,  was un-identifiable with Protocol 3. This result occurs because of the lack of distinction in the coefficients of the parameters at the sample times as indicated in Table 5 and Figure 3.

Thus, the method predicted the poor performance of the third protocol while it predicted much lower variability for both Protocols 1 and 2. However, it also suggests that Protocol 2 would improve slightly upon Protocol 1, which was not the case as both protocols performed equally in terms of parameter identifiability. It is expected that it's equality of variance is an artefact of the normalisation as a function of mean coefficient at the sample value, artificially lowering the magnitude of the ||Δ||_2 _terms in Protocol 1.

Overall, these findings highlight the inefficiency, extreme clinical burden and intensity of frequent sampling in contrast to well-positioned and infrequent sample timing. More specifically, Protocol 1 used 9 more samples than Protocol 2 with significant added clinical intensity and assay cost (^~^200% more!) for absolutely no information gain. This outcome was successfully predicted by the identifiability analysis method presented.

### 3.2 Analysis of the glucose PD model

Equation 4 will be used in the analysis of identifiability of terms frequently used to model the PDs of insulin and glucose. All analyses in this section will simulate insulin concentration profiles for the plasma and interstitium only once for each Monte Carlo analysis. Thus for clarity and simplicity, it is assumed that insulin is not subject to assay error here. Furthermore, while glucose assay error from a blood gas analyser is approximately 2%, errors of 1% and 3.5% will be used for consistency with Section 3.1. As such, the resultant coefficients of variation should not be considered fully applicable clinically, but merely as an indication of parameter trade-off during identification. The Monte Carlo analysis method with the NGT participant described in Section 2.3 is repeated for the glucose PD model. The IGT participant will be used in tandem with the NGT participant from Section 3.2.2.

#### 3.2.1 Insulin sensitivity and distribution volume

**Figure 4:**
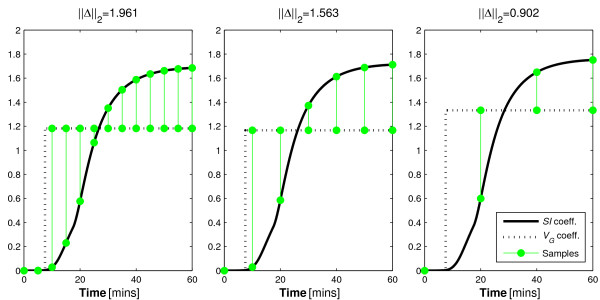
**Insulin sensitivity and glucose distribution volume coefficient integrals**. Distinction between the integral of the coefficients of the parameters of Equation 5 when differing sampling resolutions are used.

Use of the DISST model typically entails the identification of *SI *and *V_G _*in Equation 4 [18,20,25]. As such, this case is tested using the proposed method and three potential sampling protocols. Specifically, Protocol 1 uses the 5-minute sampling resolution described in Section 2.4, while Protocols 2 and 3 use 10 and 20-minute resolutions, respectively. Figure 4 and Table 7 indicate that these parameters are uniquely identifiable in the presence of measurement noise given a surprisingly small number of data points.

**Table 7:** Sample resolution dependence of identified *SI *and *V_G _*values

Noise	1%			3.5%		
Protocol	1	2	3	1	2	3
***SI***	1.001(0.007)	1.002(0.008)	1(0.016)	1.002(0.028)	1.002(0.033)	1.010(0.054)
***V_G_***	1(0.012)	1(0.014)	1.001(0.029)	0.998(0.046)	1.001(0.049)	0.990(0.087)

The normalised mean and (CV) of the glucose PD parameter values from the three proposed sampling resolutions.

Table 7 shows that parameter stability is generally very high even in a sparsely sampled data set with a relatively high level of noise. This result was expected due to the relatively large difference in the coefficient integrals shown in Figure 4 for each of the sampling protocols. Thus, like the case of *V*_*P *_and *n_T_*, intelligent sample timing can significantly reduce clinical burden and study cost with negligible loss of information. Furthermore, the proposed method successfully predicted this outcome.

#### 3.2.2 Insulin sensitivity and glucose dependent decay

Model-based studies of insulin sensitivity frequently identify a parameter synonymous with *p_G _*in addition to *SI *and *V_G _*as parameters when using the Minimal Model [10] or similar. However, although these parameters are mathematically distinct, they are known to trade-off during identification and practical identifiability is not generally assured. It has been reported that these issues can be exacerbated for insulin resistant (IR) individuals [7,8]. Thus, the second, IGT participant defined in Table 3 will also be analysed.

**Figure 5:**
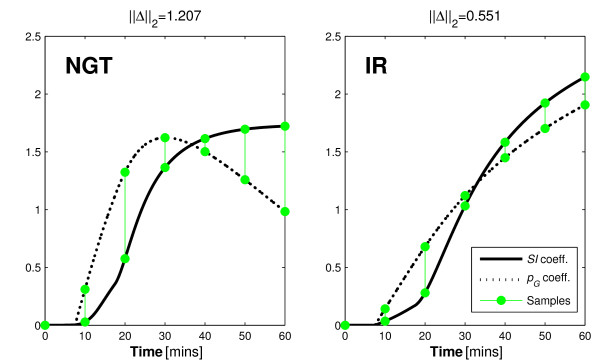
**Insulin sensitivity and glucose dependent uptake in NGT and IR individuals**. The disparity between normo-glucose tolerant and insulin resistant individuals in terms of the distinction of the integral formulations of the coefficients of *SI *and *p_G_*.

Some insight into the parameter trade-off during identification of dynamic test data can be seen in Figure 5 that contrasts the integral formulations of the parameter coefficients based on glucose tolerance status. The contrasting shape of the integral formulations of the parameter coefficients is best observed in the *p_G _*coefficient. The *p_G _*coefficient is the only term in Equation 4 that could possibly become negative. Thus, the integral of the coefficient can form a convex shape that contrasts well with the coefficient of *SI *as seen for the NGT participant in Figure 5. However, the negative coefficient of *p_G _*can only occur when the participant's glucose concentration goes below the basal concentration. Thus, as only NGT participants achieve such concentration reductions in typical dynamic insulin sensitivity tests, the parameter identifiability of IR participants is impaired in comparison. In particular, Figure 5 (right) shows minimal difference and a much smaller ||Δ||_2 _value for this IR individual indicating increasing potential for parameter trade-off in the identification process and loss of effective identifiability. This is despite an identical test protocol and model.

As mentioned, this limitation of the Minimal Model has been reported, but not explained in the literature until now.

Table 8 shows the parameter error when the 60 minute 10-minute sampling protocol is used.

**Table 8:** Participant dependence of identified *SI *and *p_G _*values

Noise	1%		3.5%	
Participant	NGT	IR	NGT	IR
***SI***	1(0.008)	1.001(0.161)	0.998(0.032)	0.876(0.449)
***p_G_***	1.009(0.320)	0.959(0.756)	1.109(0.802)	1.573(1.244)

Normalised mean and (CV) values of the identified.

It is apparent that the insulin resistant individual's parameter identifiability is much lower than the NGT participant despite the identical PD model, test protocol and identification process. Table 8 highlights this result, as well as the increasing loss of identifiability as assay error increases. This is in accordance to published findings and the proposed method's prediction.

#### 3.2.3 A hypothetical protocol to enable p_G _identification in dynamic tests

To forcibly remove this ambiguity introduced by the comparable coefficients of the IR individual, the protocol of the DISST could be altered. After an initial observation of the effect of the insulin bolus on glucose concentration, more insulin could be introduced to ensure that the participant's glucose concentration is maintained approximately 0.5 mmol/L below the basal concentration. Such a protocol may include an extension of the protocol described in Section 2.4 wherein a period of slight hypoglycaemia is achieved for each participant with a series of participant-specific insulin boluses administered with feed-back control.

To allow a fair comparison between the variability of the parameters of the proposed protocol and the protocol used in Section 3.2.2 the sampling regimen and test duration will be maintained. Thus, the additional insulin is administered as a bolus *t *= 32.5 minutes. In a clinical setting, the magnitude of the bolus would be participant-specific and dependent on the glucose concentration response alone (as glucose can be assayed in real-time). Note that this task would be very difficult and potentially dangerous in a regular clinical setting. In particular, the amount of insulin required would vary between participants, and must be estimated in ignorance of endogenous insulin production. This may cause a high incidence of potentially harmful hypoglycaemia. This protocol is only mooted to illustrate the ability of the method, and in reality the introduction of additional insulin could be applied slowly and safety would be assured.

For the case of the IR participant presented here, reducing glucose sufficiently would require a 3U bolus at *t *= 32.5 minutes.

**Figure 6:**
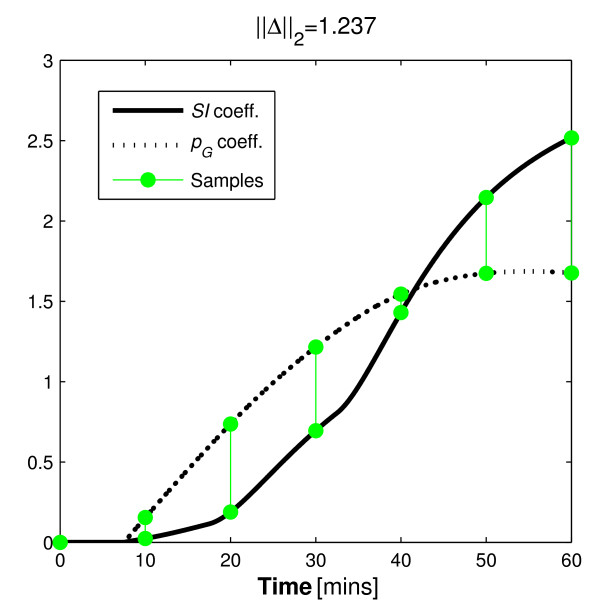
**Effect of an alternative protocol to maximise parameter distinction in IR individuals**. The effect of the added insulin bolus on the distinction of the coefficient integrals of *p_G _*and *SI *for the IR participant.

The proposed protocol would alter the shapes of the integral of the parameter coefficients for the resistant individual. In doing so, it is hypothesised that it would increase the distinction between these curves to avoid the similarity seen in Figure 5 (right) to ensure identifiability. The ||Δ||_2 _value obtained for the IGT participant and the updated protocol indicates a reduction in variability ratio of approximately 1:2.2. Figure 6 contrasts with Figure 5 (right) as it shows how the added bolus significantly increases the distinction between the coefficients of the identified parameters. Table 9 shows the outcomes of this analysis.

**Table 9:** Effect of the added insulin bolus on the identifiability of *SI *and *p_G _*from the IR participant

Noise	1%	3.5%
***SI***	1.002(0.029)	0.996(0.070)
**Δ*SI***	+0.001**(-0.132)**	+0.120**(-0.379)**
***p_G_***	0.989(0.369)	0.992(0.969)
**Δ*p_G_***	+0.030**(-0.387)**	-0.581**(-0.275)**

Parameter convergence from the proposed hypothetical protocol. Δ*SI *and Δ*p_G _*show the change between these values and those from the same individual presented in Section 3.2.2. The bold ΔCV values are the critical finding of this analysis.

Although the inhibitive CV values for the parameters indicate that the proposed protocol could not be used clinically, the variability decreased in the order predicted by the identifiability method presented. The hypothetical protocol presented has confirmed the reasons discussed for the poor parameter identification observed in many clinical studies which utilise these two competing parameters [7,8]. Furthermore, it demonstrates a limitation of traditional identifiability methods, which provide an evaluation of identifiability in ignorance of probable participant behaviour or test protocol design.

The protocol presented would be virtually impossible to apply clinically in the 60 minute duration as it is shown here. However, this analysis was limited by the need for a comparable duration and sampling regimen to Section 3.2.2. The method could thus be used to define similar protocols that could yield data that enables unique identification of these parameters. If such protocols are pursued, they would most likely require two sections in a much longer test. The first section would involve the protocol defined in Section 2.4, and would be followed immediately by an infusion of insulin designed to safely bring the participant's glucose concentration to 0.5 mmol/L below the basal level. Robust results would be most assured if the participant's glucose concentration was maintained below this level for approximately 30 minutes, and thus, the protocol would most likely require about 2 hours. However, a stable result both in terms of *SI *and *p_G _*would be generally assured.

#### 3.2.4 Removal of redundant points

**Figure 7:**
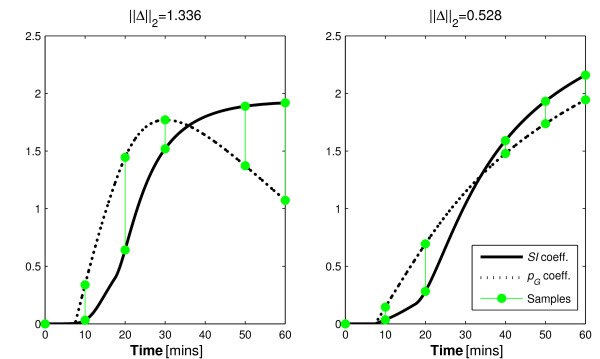
**Effect of omitting assumed negligible samples**. The coefficient comparison in alternative sampling protocols that omit the samples that according to Figure 5 have almost negligible value in terms of identifiability due to the very small magnitude of distinction at the sample times.

The *t *= 40 minute sample in Figure 5 (left) and *t *= 30 in Figure 5 (right) show virtually no distinction between the coefficients of either profile. Thus, according to the theory presented, it should provide no value to the identification process. To test this, the analysis of Section 3.2.2 is repeated with these samples removed. Figure 7 shows how the omitted data point do not significantly alter the distinction shown in Figure 5 (Section 3.2.2). Table 10 shows how the identified parameters were affected by the omission of data that the method implied were redundant.

**Table 10:** The effect of targeted sample omission

Noise	1%		3.5%	
Participant	NGT	IR	NGT	IR
***SI***	0.999(0.010)	0.960(0.182)	1(0.029)	0.852(0.496)
**Δ*SI***	-0.001**(+0.002)**	-0.041**(+0.021)**	+0.002**(-0.003)**	-0.024**(+0.047)**
***p_G_***	1.019(0.360)	1.152(0.693)	1.400(0.762)	1.231(1.156)
**Δ*p_G_***	+0.010**(+0.040)**	+0.193**(-0.063)**	+0.311**(-0.040)**	+0.342**(-0.088)**

Parameter normalised mean and (CV) values of identified parameters with the omission of the assumed negligible data points. Δ*SI *and Δ*p_G _*are the change in values between this analysis and Section 3.2.2. The bold ΔCV values are the key finding of this analysis.

The findings of this analysis imply that the omission of the samples that were assumed to be obsolete had little effect on the outcome of the identification process. Most changes were very small and only those for the particularly un-stable parameters showed any significant changes.

### 3.3 The value of μ

The value of μ in Equation 1 can be used to enable prediction of the probable variability in the identified parameters in a Monte Carlo simulation. Thus, the effects of protocol changes on the parameter identifiability can be predicted without the need for numerous Monte Carlo simulations. To identify the value of μ linear relationships between the CV values obtained and the *ε*/||Δ||_2 _values are defined. As noiseless identifiability of all models has been proven, the y-intercept can be assumed at zero, and μ can be identified using Equation 6:(6)

**Figure 8:**
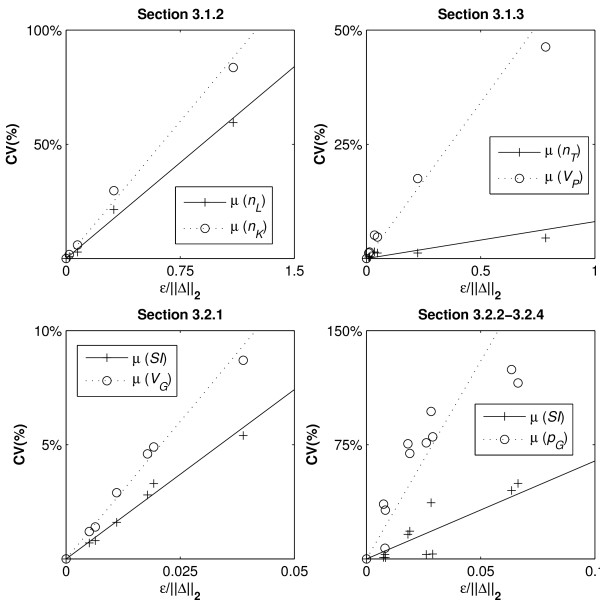
**Linear regression of μ**. Comparisons between CV values and *ε*/||Δ||_2 _terms to provide parameter specific values for μ.

Figure 8 shows the adherence of μ to linear relationships while Table 11 shows the value of μ for the different parameters.

**Table 11:** μ values from the various analyses

Analysis	Parameter	μ
**Section 3.1.2**	***n_L_***	0.560
	***n_K_***	0.801

**Section 3.1.3**	***n_T_***	0.081
	***V_P_***	0.683

**Section 3.2.1**	***SI***	1.48
	***V_G_***	2.41

**Sections 3.2.2 and 3.2.4**	***SI***	6.45
	***p_G_***	26.0

Table 11 presents the μ values identified by the gradients of the regression lines

It can be observed that no single value for μ can be applied across all models and that different parameters are considerably more susceptible to the distinction of the parameter coefficients. However, the general adherence to the linear relationships observed in most examples implies that the form of Equation 1 is accurate for this purpose with the possible exception of *SI *in Sections 3.2.2 and 3.2.4.

## 4. Conclusions

The method presented was able to predict and explain the parameter identifiability behaviour exhibited in models of insulin kinetics and insulin/glucose dynamics. This capability is in direct contrast to the traditional derivative-based identifiability analysis [1,3,11-13] that can only provide a confirmation of an infinitesimal distinction of the parameters in the governing equations. In addition to the ability to predict parameter identifiability, the method has been shown to be able to aid sample selection and explain the non-identifiability of the *p_G _*term in common insulin sensitivity tests for IR participants of dynamic tests. It was also used to derive and justify a novel protocol to make this parameter more identifiable in this subgroup. Furthermore, the method also accurately predicted sample redundancy.

Like the proposed method, the method proposed by Raue *et al. *[4] recognises the ability of apparently identifiable parameters to become practically non-identifiable in the presence of assay error and discrete sampling. The Raue *et al. *method requires numerous simulations and parameter identification processes to characterise the model sensitivity to variations in each model parameter. In contrast, the proposed method allows a quick graphical analysis, which can produce immediately apparent and intuitive results. Furthermore, the proposed method can quickly appraise protocol variants, and provide indications of the reason for practical non-identifiability. The method of Raue *et al. *is a more general method as it can appraise most model configurations, whereas the proposed method is currently limited to models with two separable parameters.

In reality, many models utilise more than two parameters to describe physiological kinetics and seek to identify all at once. It is expected that development of the proposed method will enable identifiability analyses of such models. However, more care must be taken to construct the coefficient integral formulations as combinations of parameters that may come into conflict. This task would require the contrast between the most deleterious combinations of integral coefficients to be measured. However, this point was not explored in this study, as the goal was to introduce the overall approach.

Only cases with separable parameters in terms of measured species were analysed. In reality, some model parameters are intrinsically linked and this method will not work. An example of linked parameters would be insulin sensitivity and insulin effect saturation that requires a Michaelis-Menten formulation. In addition, some parameters effect remote, un-measured concentrations or masses and are thus not able to be identified with this method. For example, the *n_C _*term in Equation 3 could not be evaluated for identifiability using this method without the inclusion of measurements of interstitial insulin [26]. As such, this method should not replace the traditional identifiability analysis, but be used in tandem with it (or methods such as Raue *et al. *[4] or similar) to produce both theoretical and practical investigations of identifiability.

Furthermore, the model assumes that an expected range of parameter values is known prior to the commencement of the clinical study. This knowledge is important, as the method requires that species simulations are available to define the coefficients of the parameters. However, in most cases, the researcher will be able to obtain an indication of the likely range of parameter values in a cohort from a brief literature search, and likely test outcomes could be cases that can be safely evaluated prior to clinical testing.

There is also an assumption that the model captures all of the dynamics of the system perfectly. In reality no model can provide such accuracy. In particular, Figures 4, 5, 6, 7 show a sample taken at *t *= 10 for glucose. In reality, this sample will be affected heavily by error caused by incomplete mixing, and although the method presented indicates that this is a valuable sample, if it is used in the glucose pharmacodynamic model of Equation 5, the resultant parameters will be overly influenced by an un-modelled mixing effect.

Although the method has limitations and potential for improvement, it can provide valuable information at a study design stage, as well as valuable identifiability information not available from typical methods. It can differentiate between the applicability of different dynamic tests based on cohorts, and also help to define optimal sample timing and frequency. In particular, it explained the observation of poor parameter convergence in the Minimal Model for insulin resistant participants that has been widely reported but without clearly defining the cause. Thus, despite the method's limitations, it should still be used in the design stage of a study to ensure that the resultant clinical data can provide usable results, and time and money is not wasted.

Finally, the method has highlighted the limitation of discrete binary identifiability analyses as providing potentially misleading assurances of parameter identifiability in real clinical applications, and shown that identifiability is instead a continuous artefact of sample timing and the distinction between parameter coefficients.

## Competing interests

The authors declare that they have no competing interests.

## Authors' contributions

PDD contributed to the development of the concept, investigation design, interpretation of results, and drafted the manuscript. JGC and TD refined the analysis and interpretation, and edited the manuscript. TFL contributed to the investigation design, interpretation of results, and edited the manuscript. All authors have read and approved the final manuscript.

## Appendix 1: Structural identifiability

### A1.1 Structural identifiability of the insulin model

Using the method of algebraic derivative approach of [21] and refined in [1] the identifiability of the model can be confirmed. Using the ranking:

generates the characteristic set of Equations 2 and 3.(3a)(2b)

if: *Y *= *I*

Thus, the following coefficients can be defined:

Using arbitrary values for the coefficients confirms global identifiability of the insulin kinetic model of Equations 2 and 3.

### A1.2 Structural identifiability of the glucose model

The structural identifiability of the glucose model is considerably less complex than the insulin kinetic model. In particular, the characteristic set is defined:(4a)

and thus the coefficients are defined:

and observation confirms that *p_G_*, *SI*, *G_b _*and *V_G _*are globally identifiable using derivative-based identifiability analysis.
